# DAD-match; Security technique to prevent denial of service attack on duplicate address detection process in IPv6 link-local network

**DOI:** 10.1371/journal.pone.0214518

**Published:** 2019-04-02

**Authors:** Ahmed K. Al-Ani, Mohammed Anbar, Selvakumar Manickam, Ayman Al-Ani

**Affiliations:** National Advanced IPv6 Center (NAv6), Universiti Sains Malaysia (USM), Gelugor, Penang, Malaysia; Victoria University, AUSTRALIA

## Abstract

An efficiently unlimited address space is provided by Internet Protocol version 6 (IPv6). It aims to accommodate thousands of hundreds of unique devices on a similar link. This can be achieved through the Duplicate Address Detection (DAD) process. It is considered one of the core IPv6 network’s functions. It is implemented to make sure that IP addresses do not conflict with each other on the same link. However, IPv6 design’s functions are exposed to security threats like the DAD process, which is vulnerable to Denial of Service (DoS) attack. Such a threat prevents the host from configuring its IP address by responding to each Neighbor Solicitation (NS) through fake Neighbor Advertisement (NA). Various mechanisms have been proposed to secure the IPv6 DAD procedure. The proposed mechanisms, however, suffer from complexity, high processing time, and the consumption of more resources. The experiments-based findings revealed that all the existing mechanisms had failed to secure the IPv6 DAD process. Therefore, DAD-match security technique is proposed in this study to efficiently secure the DAD process consuming less processing time. DAD-match is built based on SHA-3 to hide the exchange tentative IP among hosts throughout the process of DAD in an IPv6 link-local network. The obtained experimental results demonstrated that the DAD-match security technique achieved less processing time compared with the existing mechanisms as it can resist a range of different threats like collision and brute-force attacks. The findings concluded that the DAD-match technique effectively prevents the DoS attack during the DAD process. The DAD-match technique is implemented on a small area IPv6 network; hence, the author future work is to implement and test the DAD-match technique on a large area IPv6 network.

## Introduction

**Fig 1:**
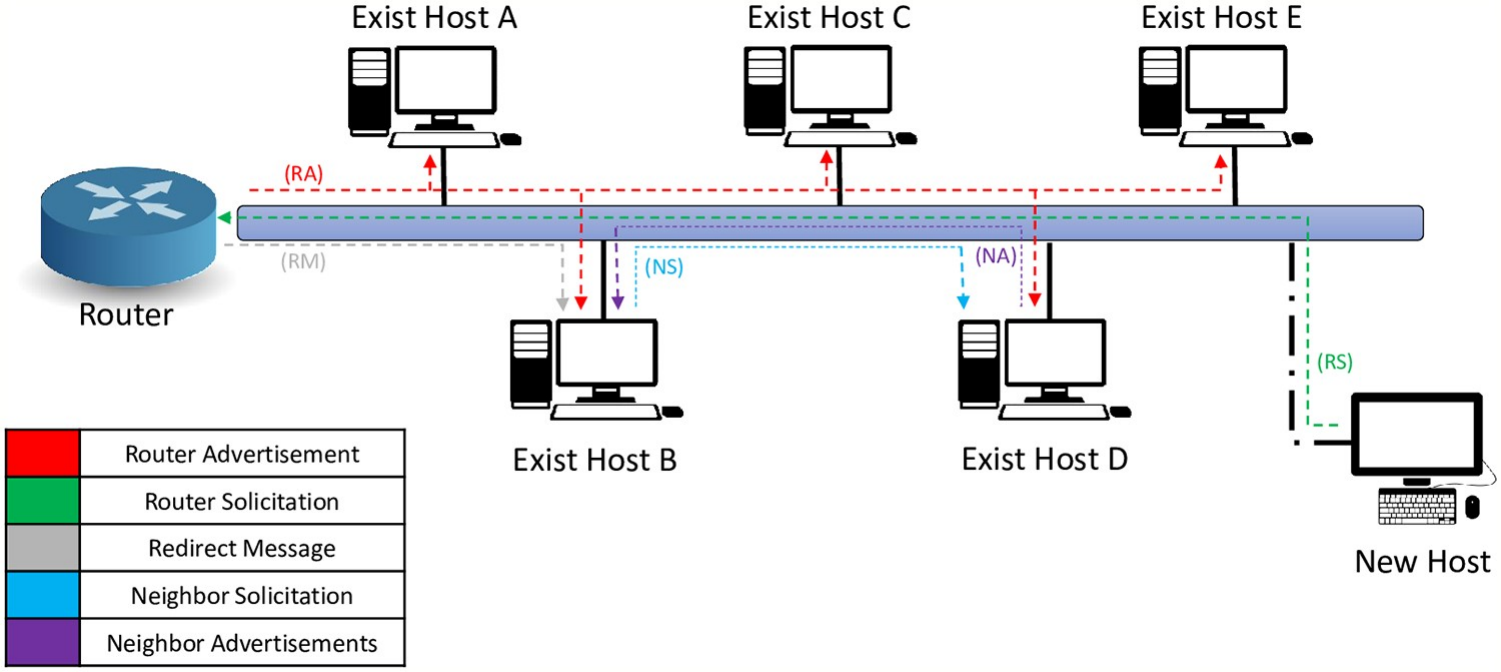
NDP messages.

IPv6 was defined amid the 1990s in Request For Comments (RFC 2460) [1]. It aims at extending and ultimately replacing the ability of IPv4 with expectations that it would help improve various aspects of IPv4, address challenges, and more significantly secure the Internet [2]. It enjoys novel features, which include extended addressing capabilities to 128-bits using a simpler header format [3]. It introduces a new concept called ‘Neighbor Discovery Protocol’ (NDP), which aims at boosting the network’s communication, making it faster, and extending it to reach out for a large area. NDP [4] uses five of the Internet Control Message Protocol version 6 to provide several functions like addressing resolution to request another host link-layer address, Neighbor Unreachability Detection (NUD) to detect reachable hosts, auto-configuration for generating an IP address automatically, and the DAD process to ensure that there is no IP conflict on the same link. Fig 1 illustrates the five NDP messages.

NDP is considered as an essential protocol in IPv6, which does not have a sufficient security strategy to offer verification and authentication of the packets exchange among the hosts that are located on a similar link. According to previous scholarly studies [5], [6] it is proven that the local network’s communication neighboring hosts are not totally reliable because they are more likely to act as attackers. Researchers [7], [8] investigated dangers and weaknesses of neighboring discovery. The attackers can take advantage of vulnerabilities in the neighboring discovery to launch a DoS attack. This makes the network’s performance less competent and leads to traffic hijack.

The DAD process is one of NDP functions. It permits the node to configure a unique IP after confirming it with the present hosts on a similar link. Any node on a similar link can expose the DAD process to the DoS attack by responding to each NS message, which is transmitted from the target host since NDP does not have a security mechanism to secure messages, that is, (NS and NA messages). Ultimately, the IPv6 address network interface cannot be configured by the target host. This prevents it from joining the IPv6 network. RFC (Request for Comment) 4861 recommends the use of two security methods: IPSec [9] and SeND [10] for protecting NDP functions, which include DAD. Utilizing the two proposed methods, however, is challenging as IPSec undergoes bootstrapping in case it is utilized for NDP as investigated by [11]. The SeND method, on the other hand, draws on a complicated algorithm that requires heavy computation, which demands huge time and resources’ consumption [11]. In addition, SeND complexity can be targeted by a DoS attack.

A study by [12] has introduced a new technique for the DAD process security in IPv6 link-local network using a SHA-3 hash function to hide the tentative IP address. The new security technique is called ‘DAD-match’. The DAD-match security technique implementation is investigated in this research paper. Moreover, an evaluation of the obtained results with other existing techniques is carried out. The paper is organized into nine sections. The first section introduces the topic under investigation. The second and third sections give an overview of the DAD process along with security challenges. Relevant scholarly works on how to secure DAD are reviewed in Section 4. Section 5 presents and discusses the proposed DAD-match technique. A comprehensive analysis is given in Section 6. Experiments and evaluation, as well as discussion, are given in Section 7 and Section 8, respectively. Section 9 puts forward conclusions, recommendations, and aspects of further research.

## Overview of duplicate address detection

Duplicate Address Detection (DAD) refers to a process, where distinctiveness of all configured interface identifiers (IID) (e.g., for IP addresses within a similar link) is guaranteed. In a normal situation, a host works on performing DAD before it assigns a certain address to an interface [13]. In case a new host is connecting to an IPv6 local link or an existing host on a similar link is generating a new IP address with a previously defined prefix of FE80:: and 64 bits IID, the new IID is formed by either Extended Unique Identifier (EUI-64) [1] or privacy extension [14] method. The difference between both methods is that EUI-64 is an address format. It is obtained on Ethernet interfaces through referencing the already unique 48-bit MAC address and reformatting the value so that the EUI-64 specification matches. This technique, however, generates a similar IID when the network is joined by a node and, therefore, it makes it easy to track the node by intruders. Privacy extension method, on the other hand, refers to the technique, through which an IP address is generated in a random way [14]. Based on the privacy extension method, the address keeps changing as time goes by and, therefore, it makes it hard to identify the target host address by intruders and other eavesdroppers [15].

**Fig 2:**
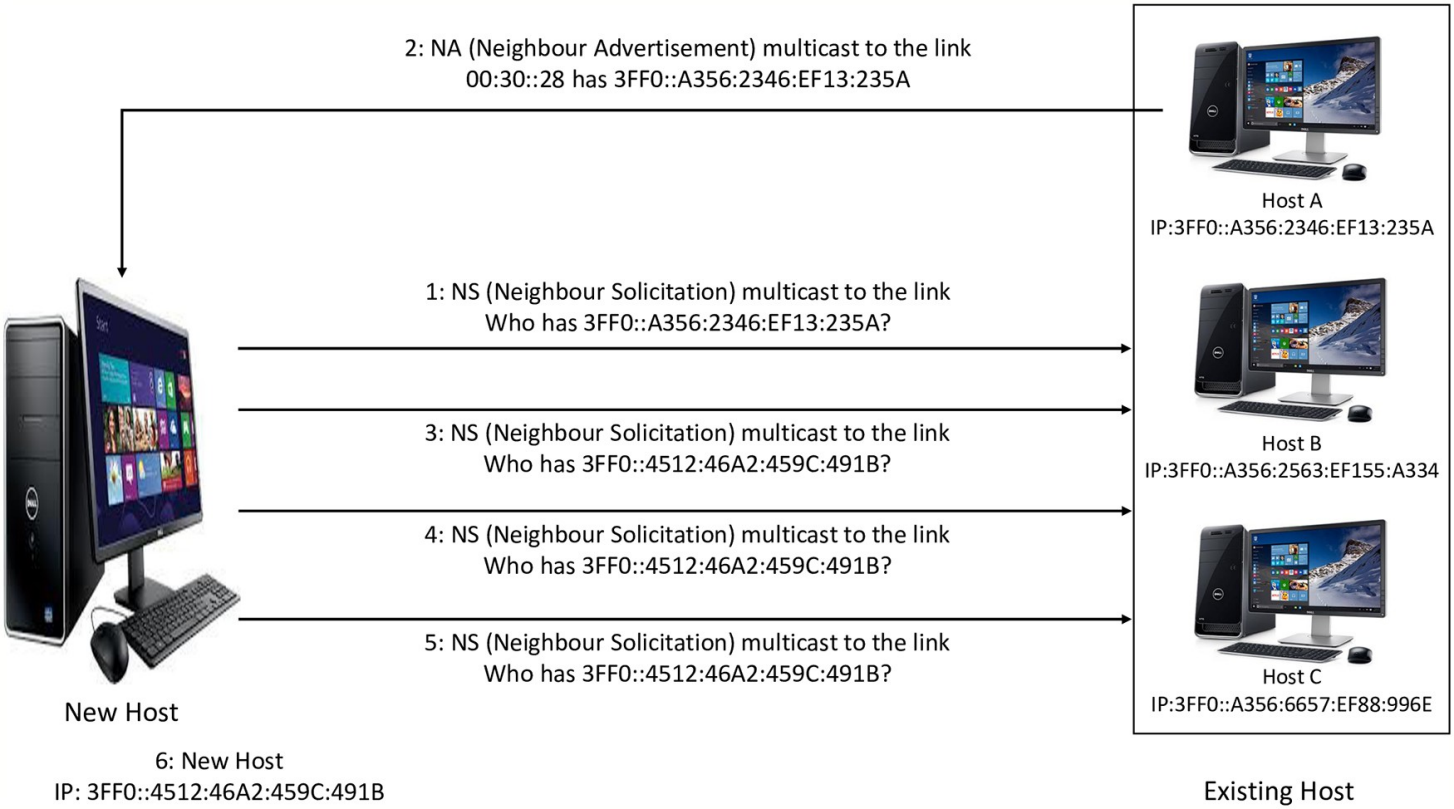
DAD process in IPv6 link-local network.

After an IP address is generated, it becomes a tentative IP address because its uniqueness has not been confirmed yet. Therefore, the target host verifies its uniqueness (that there is not any existing neighbor host on the same link using the same generated IP) via the DAD process using two of the NDP messages (NS and NA messages). Fig 2 shows the DAD process in IPv6 link-local network.

The target host should multicast the NS message, which carries the tentative IP address to Solicited-Node Multicast Address (SNMA) FF02::1: FF:00:0/104 (based on the last 24bit from the tentative IP) within an unspecified source address (::) to target the unverified address. If another neighbor node occupies the generated IP address, the NA message is sent in the form of a reply. If the NA message is not received or in case the NA message is not received after 3 seconds, the processing host interprets such a situation as there is no matching IP address on a similar link. Accordingly, a host uses that generated tentative address as a preferred address. Furthermore, after running 3 trials, the new host puts the DAD to an end before making its network interface assign an IPv6 address [16].

## Denial of service attack on duplicate address detection (DoS-on-DAD)

Generally speaking, the existing hosts in IPv6 link-local network are dependable (i.e., any of them can participate in DAD). An ICMPv6, which is also known as an NDP message, is employed during DAD to facilitate NS and NA verification [17]. Because NDP messages by default are unsecured [16], any active intruding host can, therefore, benefit from the unsecure NDP design by using DAD via manipulating ND messages. Attackers can interrupt and negatively affect verification by sending bogus reply messages when a new host starts DAD. Researches [8], [16], [18] emphasized that DAD is vulnerably exposed to DoS attacks.

**Fig 3:**
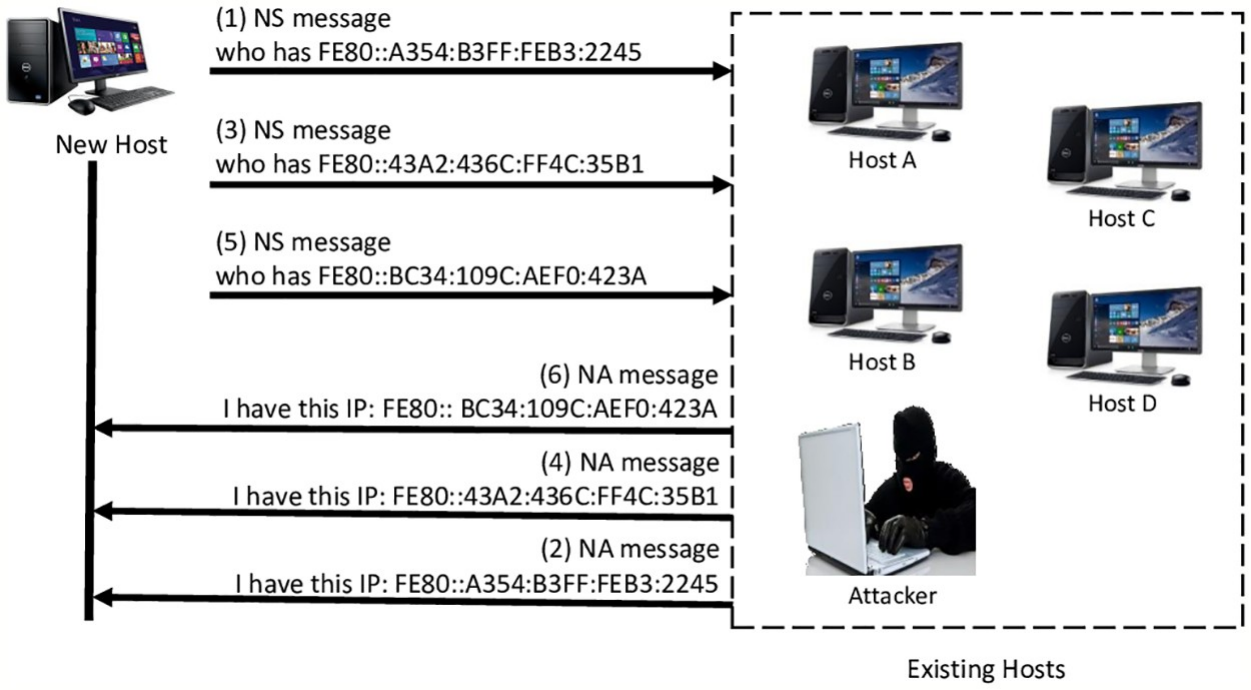
DoS on DAD process in IPv6 link-local network.

DoS-on-DAD attack prevents a target host from obtaining an IP address by constantly sending NA messages to claim that a generated IP is not unique. Consequently, the target host is unable to join the network and communicate with other existing neighbor hosts due to the DAD process failure [19]. Fig 3 shows DoS attack on DAD process.

New hosts, for example, transmit NS messages to existing hosts throughout the DAD process so that a tentative IP address uniqueness is substantiated. By providing the tentative IP address via a bogus NA message, the attacker responds. A tentative IP address cannot be regarded as unique if an NA message is received by the new host. In attacks, a new IP address is created by a new host and an NS verification message is sent. The verification is simulated by the attacker through sending the NA message. After three attempts are carried out, the new host cannot configure its IP address because of the DAD procedure failure. Therefore, the new host cannot establish communication with the existing neighboring hosts on a similar link. Fig 3 exhibits the DoS attack on the DAD process in IPv6 link-local network.

## Related work

Several mechanisms have been proposed by researchers drawing upon varied methods aiming at securing the DAD process in IPv6 link-local network. Relevant scholarly studies based on hash function and their drawbacks are critically reviewed in this section.

### Secure Neighbor Discovery (SeND)

SeND adds new options to NDP like cryptographically-generated address (CGA), Rivest–Shamir–Adleman (RSA) cryptosystem signature, time stamps, and the Nonce to secure NDP messages. It also introduces two innovative types of ICMPv6 messages. They are certificate path solicitation and certificate path advertisement [10]. SeND aims at preventing NS and NA spoofing, NUD failure, DAD DoS and NDP DoS attacks [20]. However, previous studies [8], [11], [20] highlighted the deficiencies of SeND for NDP in IPv6 link-local network. In general, security options (i.e., CGA and RSA) are limited. The CGA option cannot identify the character of a legitimate node. Therefore, the attacker node can change CGA parameters in NDP messages. As a result, an attacker can abuse target hosts during NDP. Another limitation of SeND is related to the basic RSA signature design, which adds a considerable amount of process overheads. Given that CGA and RSA are the primary components, SeND requires sizable processing time (CPU and bandwidth of IPv6 hosts) and, therefore, increases complexity during NDP in IPv6 link-local network. Malicious hosts can exploit SeND when IPv6 hosts are engaged in a neighboring, discovery–message verification process. Consequently, the DoS attack against the targeted host may occur during DAD in IPv6 link-local network.

### Trust—Neighbor Discovery (Trust-ND)

Trust-ND protects the IPv6 communication including the DAD process by securing NDP messages [6]. The SHA-1 hash function is utilized to build up a mechanism that is used for achieving the required security [21], [22]. It introduces a novel security opportunity known as Trust-Option, which is attached to each NDP message (i.e., NS and NA messages) so that communication among hosts is established. It has been argued by some research groups that Trust-ND provides weak security for IPv6 DAD. Others studies [18], [23] indicated that the SHA-1 hash functions are vulnerably exposed to hash collision attacks. Accordingly, any hostile host can cause hash collision attacks against SHA-1 [24]. Given that Trust-ND depends on the SHA-1 hash function to establish security, Trust-ND is vulnerably exposed to collision attacks and resultant DoS attacks during DAD in IPv6 link-local network. Therefore, by design, Trust-ND cannot be considered as an appropriate technique for IPv6 DAD to establish security.

### HASH-secure target address in DAD (HSEC-Target-DAD)

A recently conducted study in 2018 by [25] introduced a new mechanism to secure target address “tentative IP address” by using a hybrid method, that is, (SHA-512 and RSA). Each node should generate public and private keys. Before sending any NS messages, the node should hash the tentative IP address by SHA-512, then extract the last 64-bits and encrypt it using the private key. The result should be later inserted into a HASH-TARGET-64 option and attached to the NS message, then multicast the NS message with a public key to existing nodes. The receiving node should use the public key to decrypt the message and compare the hash. In case the receiving node wants to send an NA message, it should use its own private key and multicast the public key to the target node.

Studies [26], [27] showed that the RSA algorithm requires a bigger key length for a higher security level. It is slow, especially for keys’ generation. Moreover, extracting only 64-bits of hash function increases the probability of a hash collision attack. The proposed mechanism sends the public key in the NS message as a text, thus may the attacker use the public key and generate a fake NA message. Additionally, HSEC-Target-DAD places the MAC address for any forged NA message in a blacklist. Based on this, the attacker may keep using other legitimate nodes MAC addresses and keep sending fake messages. Accordingly, the mechanism will block all the legitimate MAC addresses. Hence, HSEC-Target-DAD mechanism is failed to secure DAD process in IPv6 link-local network [28].

Table 1 summarizes limitations of all the existing mechanisms that proposed to secure DAD process in IPv6 link-local network.

**Table 1:** Summary of Defense Mechanisms.

Proposed Mechanism	Limitations
**SeND**	High computational cost especially for CGA and RSA option. It cannot identify the CGA address is used by legitimate node. CGA could not be used on static address configuration. High complexity. Increase network overhead and bandwidth utilization.
**Trust-ND**	Susceptible to collision attacks due to its design. Mechanism is not standardized. Vulnerable to DoS attack. Lacks addressing legitimate resource attacks.
**HSEC-Target-DAD**	High processing time during NS and NA messages generation. Vulnerable to hash collision attack. Vulnerable to DoS attack.

## DAD-match technique proposal to secure IPv6 DAD process

The DAD-match technique’s key objectives are elaborated in this section. Major issues with the existing mechanisms are discussed and justified. The suitable hash function algorithm for the DAD-match technique and the method of generating the tentative IP address are discussed. In addition, the DAD-match technique process is introduced with its verification process during the DAD process in IPv6 link-local network.

### Design goal of DAD-match security technique

It was found that the existing techniques have unsuccessfully secured the DAD process in IPv6 link-local network because of issues pertaining to genuine constraints. Therefore, a novel security technique is proposed through redesigning the DAD process to overcome the current security mechanisms’ constraints and limitations so that efficient security for the IPv6 DAD procedure is established.

The major problem with the current security mechanisms is the complexity to generate and verify the NS and NA messages as they require external resources to process them. Therefore, the CPU is consumed, as well as the bandwidth of the target node. The NS and NA messages are insecure by design and disclose the tentative IP address to public permit all the nodes on a similar link. This includes malicious nodes to join DAD and disturb the process by claiming that the tentative IP address is taken, which prevents the victim (target host) from configuring its IP address. As a result, the node cannot join the network and the DoS attack has happened. The study presumed that the DoS attack can be successfully disallowed when the tentative IP address is hidden. In addition to achieving the basic function of the DAD procedure, the design objectives of the DAD-match security technique are outlined as follows:

- Hide and secure the tentative IP address during the DAD process based on Cryptographic Algorithms.
- Secure the NS and NA messages without jeopardizing the original structure to security challenges.
- Prevent the DoS attack on the DAD process in IPv6 link-local network.

### Hash function algorithms

There exist two ways to hide the exchange tentative IP address between hosts (the target host and the existing hosts). These include encryption and cryptographic hash functions. Based on a study by [29], it is reported that encryption may introduce a heavy calculation. However, studies [7], [30] have concluded that using hash function is appropriate to meet the requirement. In comparison with encryption cryptography, it has less computation regarding processing time and much lightweight. Furthermore, the hash function algorithm can be considered strong with the following properties:

- Resistance to a Collision Attack: two inputs x and xˈ have a similar hash such that h(x) = h(xˈ).
- Resistance to a Second Pre-image Attack: given an x, finding an xˈ (≠x), such that h(x) = h(xˈ).
- Resistance to a Pre-image Attack: given an output y, finding an x, such that h(x) = y.

Many hash functions have been proposed like MD5, SHA-1, and SHA-2. However, these hash functions are vulnerable to hash collision attacks as mentioned in [18], [31]. In another study [32], it was found that SHA-2 hash value is much larger than MD5. Therefore, this long string value takes up more space and it can be slightly longer to calculate. It was revealed in that SHA3 is stronger among all the hash function proposals. Furthermore, as mentioned in, SHA-3 is a new promising generation of SHA, which utilizes fast sponge construction to generate hash values resulting in speed advantages. It has an arbitrary output length, which is different from traditional hashes in use today. It enjoys amazing security strength levels against attacks and it is flexible for implementation options for performance and security trade-offs [33].

Accordingly, the SHA-3 hash function represents the most suitable algorithm for the proposed technique as it can provide fast processing for hashing and offer availability, i.e., functionality. Moreover, it can resist different attacks like collision attacks.

### Tentative IP address generation

Any new node must generate a new IP address as a tentative IP address (128 bits), which comprises 64-bits for the network prefix. The remaining 64-bits are for the interface ID. Two common methods are used to generate the interface ID: an extended unique identifier (EUI-64) and privacy extension. IEEE defined the Extended Unique Identifier (EUI-64) as described in RFC 4291. EUI-64 utilizes a client’s 48-bit Ethernet MAC (Medium Access Control) address and inserts another 16-bits in the middle of the 48-bit MAC address so that a 64-bit interface ID is created. This method derives the IP address from the MAC address, which allows the attacker to easily estimate the IP address and facilitate DoS attacks. Additionally, EUI-64 causes privacy concerns among many users because the packets of the nodes can be readily traced to the actual physical computer and the nodes can be easily identified among the networks or across renumbering. Given these concerns, EUI-64 is unsuitable for DAD-match.

Privacy extension [14] randomly generates an IP address to prevent the attacker from predicting the IP address. This method protects users from being tracked and prevents attacks pertaining to privacy issues. The DAD-match technique, therefore, adopts privacy extension to generate a tentative IP address to maintain privacy and tackle security issues.

### DAD-match technique process

**Fig 4:**
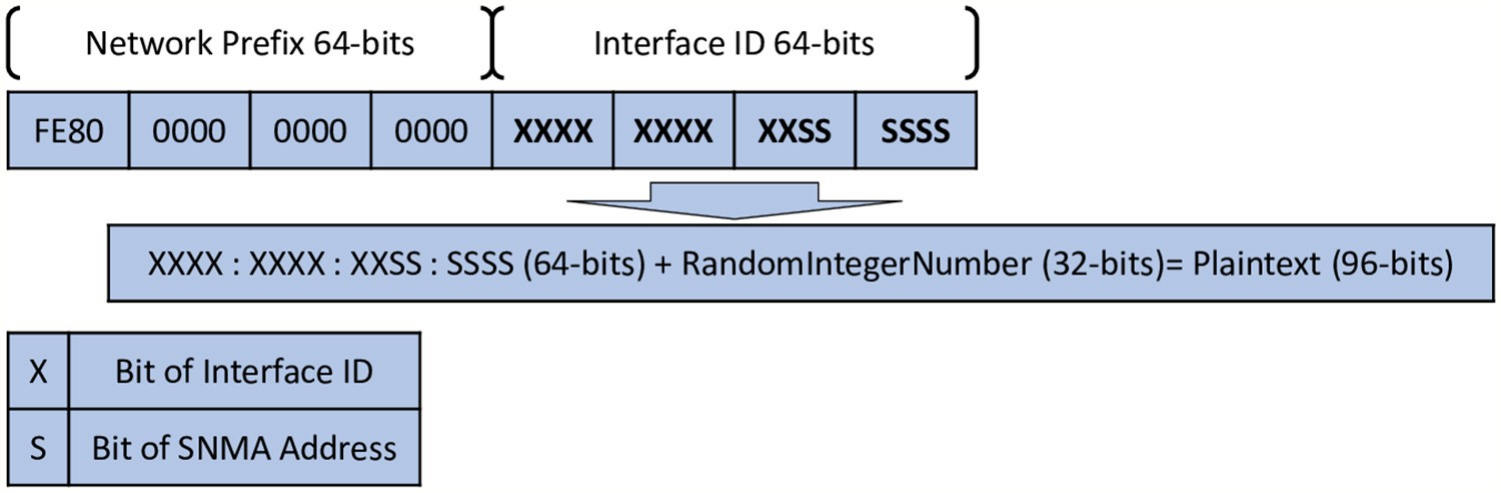
Plaintext combination.

As mentioned above, each IPv6 node, which needs to join the IPv6 link-local network, should generate an IP address and perform the DAD process on it to ensure its uniqueness. In DAD-match technique, the node must generate the tentative IP address based on Privacy Extension method instead of the EUI-64 for security issues. After that, SHA-3 (Shake128) should be applied to the Interface ID (64-bits) of a tentative IP address. Using only 64-bits, an input (plaintext) can be an issue for a brute-force attack, which uses a set of pre-defined values to attack a target and analyses the response until it is successful [34]. Accordingly, using a random integer number with 64-bits renders the plaintext difficult to predict and, therefore, launching a brute-force attack on the hash function would be hard. Fig 4 shows the combination of plaintext.

**Fig 5:**
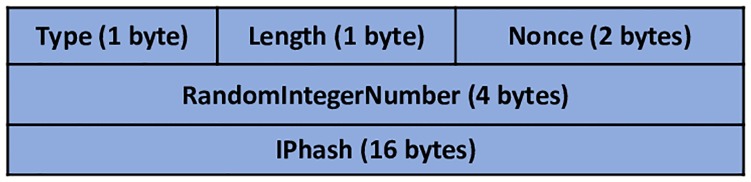
DAD*match* option format.

After SHA-3 is applied to the plaintext, the output, which is called hash values, should be carried by NDP messages (NS and NA messages) during the DAD process. DAD-match technique introduces a new option, that is, ‘DAD*match*’ for this mission. To maintain the original structure of the NDP messages, the DAD*match* design follows the option format of RFC 4861 [35]. Type and Length fields should be included in all NDP options. Therefore, the proposed DAD*match* option comprises type and length fields. The NDP option length should be 8 bytes (64-bits) to a minimum. Otherwise, the option must be padded. Furthermore, the DAD*match* option comprises 24 bytes. They are divided into five fields as follows:

- *Type*: 1-byte identifier, which indicates the option type carried by the NDP message. The DAD*match* option type is 253 because this option is used for experimentation.
- *Length*: 1-byte field to indicate the total length of the DAD*match* option. This includes the fields of type and length in 8 bytes (64-bits) unit. The DAD*match* option total length is 24 bytes and, therefore, the length field value is 3.
- *Nonce*: 2-byte field, considering that ND messages are in the form of request and response (NS and NA messages). Therefore, Nonce option or sequence number can be appropriately used to make sure that a replying message is for the corresponding solicitation message only. The function of the Nonce option aims to ensure that an advertisement is a fresh response to a solicitation request, which is sent earlier by the host.
- *RandomIntegerNumber*: 4-byte field, generating a random integer number (length value can range from 0 to 2^32) to serve as an input with 64-bits and, therefore, making it difficult for an attacker to break it. This field holds the generated random integer number, which will be used later by the receiver side for verification purposes.
- *IPhash*: 16-byte field, which carries the hash value of 64-bits and random integer number after applying a SHA-3 hash function to be used for verification by matching the hash values between the sender and the receiver. This process identifies whether the message is generated by a legitimate or fake host. This field is the main field of the DAD*match* option. Fig 5 shows the DAD*match* option format.

Combining these fields will form a DAD*match* option. It should append onto each NS and NA messages to become NS-match and NA-match messages. The verification process will be carried out on two nodes, the Receiver node and the Sender node as explained below.

### Generating and verifying DAD-match technique messages

The process of DAD-match security technique permits all the IPv6 nodes to verify the NS-match and NA-match messages whether they come from legitimate or illegitimate nodes according to the host verification response. The process of DAD-match can be divided into two parts:

### Generating and multicast the NS-match message and verifying the NA-match message

When the target host (sender) performs the DAD process, it should first generate the NS-match message with DAD*match* option including their fields: Nonce has a sequence number value, RandomIntegerNumber has a random integer number and IPhash, which has the output based on this Eq (1):

IPhash=hash(TentativeIPaddress+RandomIntegerNumber) (1)

Where, *IPhash* is the hash calculation output, *hash* is SHA-3(Shake128) hash function algorithm, *RandomIntegerNumber* is a random integer number; its values can be (0–32)-bits and *Tentative IP address* is the 64-bits of interface ID from the tentative IP address.

After the DAD*match* option is generated, it should be appended into each NS message to become NS-match message and multicast the NS-match message to SNMA address based on the last 24-bits of the tentative IP address. After that, NA-match message will be received by the target host in the form of a response towards its NS-match message. The new host should first check the DAD*match* option existence and perform the computational hash. Based on the computational hash and if the result is matching, a duplicate address takes place and the target host should re-perform DAD. Otherwise, the NA-match message is considered illegitimate by the target host. The message should be discarded and a unique IPv6 link-local address is configured. If there is no NA-match message is received after 3 seconds, the tentative IP address should be considered as unique by the target host as none of the existing hosts is using it.

#### Verifying the NS-match message, generating and multicasting the NA-match message

**Fig 6:**
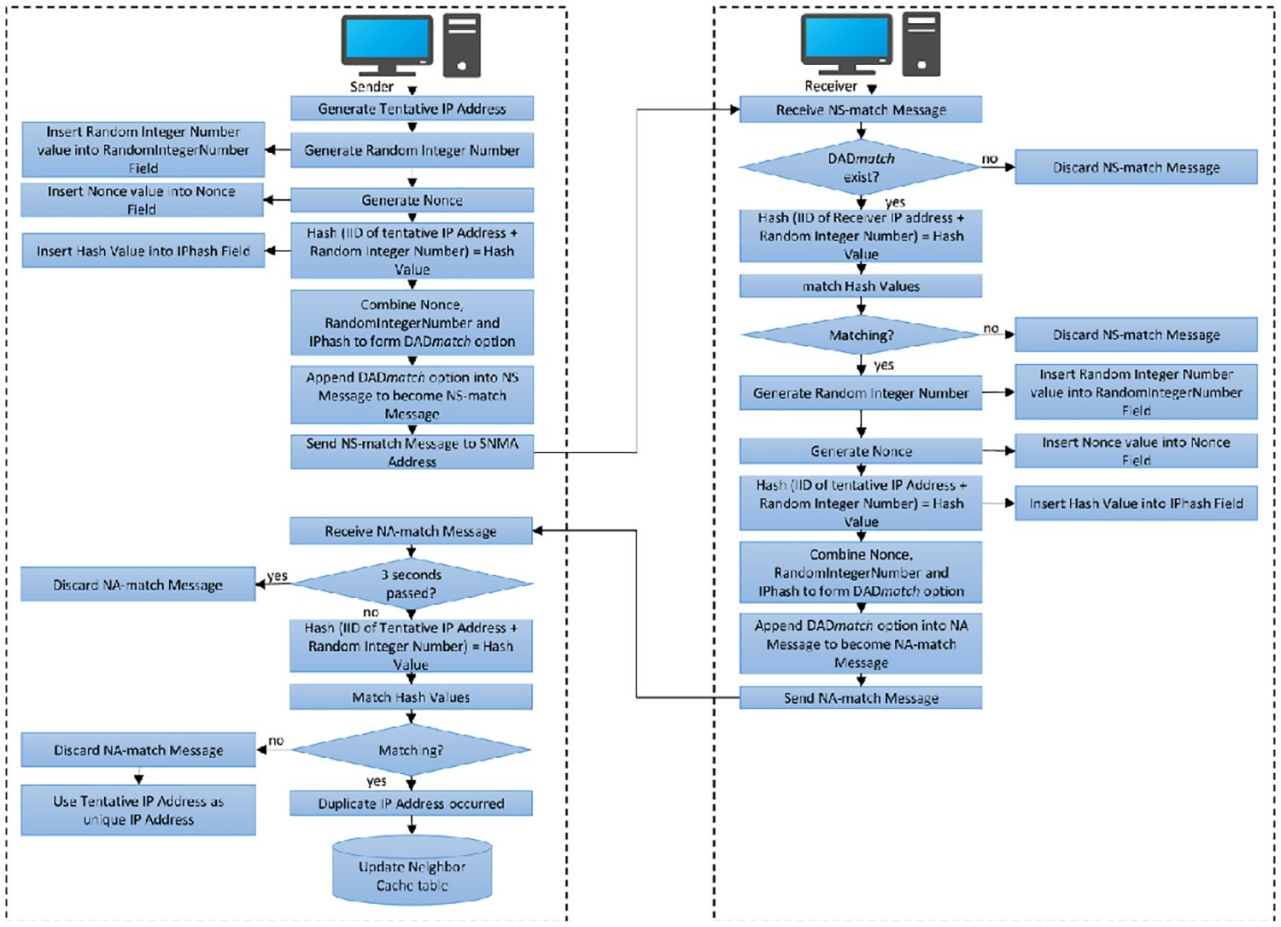
Workflow of DAD-match security technique.

After the NS-match message multicast to SNMA address, all the existing hosts that have a similar SNMA address will receive the NS-match message. The existing hosts should verify the NS-match message by checking the DAD*match* option existence, checking the nonce values, performing the computational hash by using their Interface Identifier IID (64-bits), and matching hash values. In case the hash value is matching, it will perform DAD and can reply via the NA-match message. Fig 6 shows the workflow of the proposed security technique DAD-match.

## Analysis of proposed DAD-match security technique

### Security analysis

#### Security analysis of tentative IP address

The tentative IP address represents the important information in DAD process in IPv6 link-local network. In standard DAD, the new node multicasts the tentative IP address by NS messages in plaintext. All the nodes will, therefore, receive NS messages including the attacker. Since the attacker can obtain the tentative IP address, it can claim that this tentative IP address is not unique by sending fake NA messages, which prevents the new node from joining the IPv6 network.

In the DAD-match process, the tentative IP address can be hidden using a cryptographic hash function. In this case, the attacker will not be able to obtain the tentative IP address and perform its attack.

#### Security analysis of probability of collision attack

If bandwidth *BW* on the network is 10 Gbyte, the messages *Mz* size for NS-match and NA-match is 102 bytes and the time *T* is 3 seconds, Eq (2) will calculate how many messages *Nm* the attacker can send at most:

$$N=\frac{BW}{Mz}*T$$ (2)

$$Nm=\frac{10*\;2^{30}}{102}*3\;second=315,806,418$$

Where (315, 806, 418) is the number of messages that can be sent by the attacker on the LAN within three seconds. Eq (3) illustrates the probability of collision attack *P*, where *n* is the number of bits of plaintext:

$$P=\frac{Nm}{2^{n}}$$ (3)

$$P=\frac{315,806,418}{2^{96}}=3.9*10^{-21}\;\%$$

Accordingly, the successful collision attack is ignored in DAD-match security technique.

#### Security analysis of brute-force attack

In brute force attack, the attacker tries to get all probability to break the hash within 3s. Let us assume that computer(s) are used to their full computing power for hashing, with a total of *U* CPU(s), each with *Nc* core(s) running a frequency *Fr* (in Hertz), for total time *t* (in seconds), to hash *M* messages each *b*-byte, with each hash produced requiring *Cy* cycles of one execution thread of one core of one CPU. Therefore, the *t* need can be derived from Eq (4) below:

$$T=\frac{M*Cy}{U*Nc*Fr}$$ (4)

Where the values of *Cy* cycles are obtained from SHA-3 benchmark indexed by machine. Five different machines are selected in this study to calculate the time needed for a brute-force attack on DAD-match security technique. Table 2 illustrates the machines’ details:

**Table 2:** Machine details.

Machine No.	CPU Release Date	Architecture	CPU; Cores in machine x MHz	Cycles
**1**	2017	amd64; SL+512x2 (50654)	Intel Xeon Gold 6130; 32 x 2100	930
**2**	2017	amd64; SL+512x2 (50654)	Intel Xeon Gold 6150; 18 x 2700	940
**3**	2016	amd64; BW+AES (406f1)	Intel Xeon E5-2609 v4; 8 x 1700	1584
**4**	2015	aarch64; Cortex-A57 (418fd071)	NVIDIA Tegra X1; 4 x 1734	1679.04

Based on Table 2 and Eq 3, the time needed for a brute-force attack for machine 1 is:

$$T=\frac{2^{96}*930}{1*32*2100*10^{9}}=35,251,452,470\;years$$

**Fig 7:**
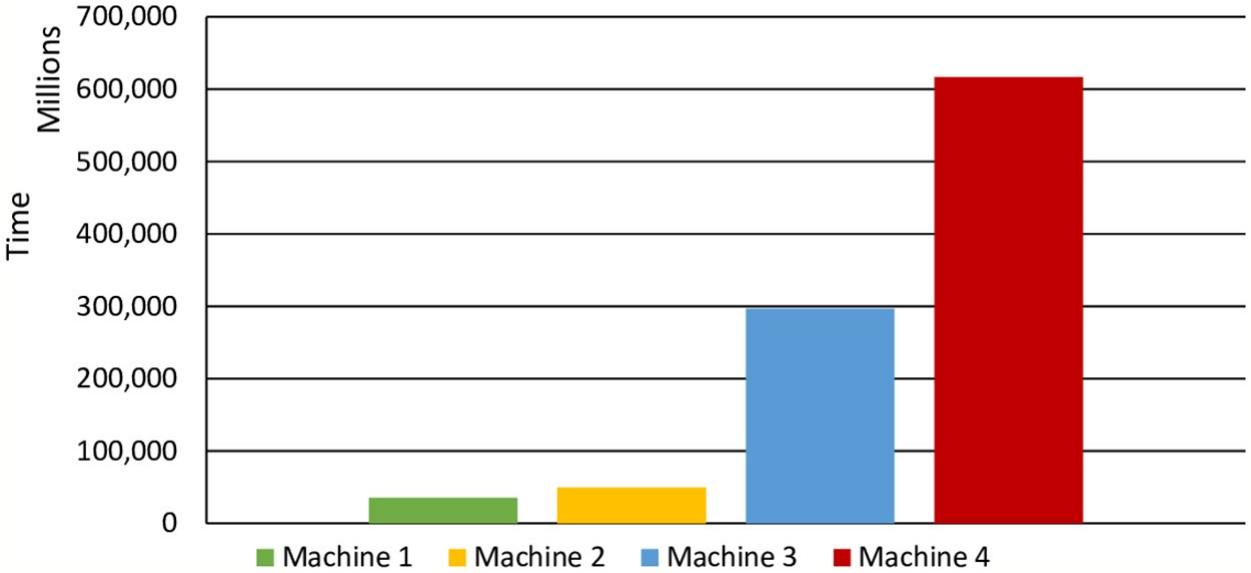
Time need for Brute-force attack.

Therefore, a brute-force attack against DAD-match technique is impossible. Fig 7 shows other machines’ brute-force attack time.

#### Network overhead analysis

To measure the performance of the proposed technique DAD-match on the network with multiple machines existed on the same network. Assumed there are 10 machines existed in the network and there is a new machine wants to join the network. In the proposed DAD-match security technique, the NS-match message should multicast to SNMA address based on the last 24-bits of tentative IP address. Thus, the probability of existing machines that will receive the NS-match message are low. To calculate the probability of machines that have the same SNMA address, can calculate by using the below Eq (5):

$$Probability=\frac{N_{m}}{2^{24}}$$ (5)

Where, *Probability* is the probability of machine has the same SNMA address and *N*_*m*_ is the number of the machine exist in the link. In the experiment there are 10 machines exist in the IPv6 link:

$$Probability=\frac{10}{2^{24}}=5*10^{-6}\;\%$$

Thus, the probability of exist machine has the same SNMA address in the link is 5 * 10^−6^, which is insignificant. Therefore, using DAD-match security technique does not cause any overhead on the IPv6 network.

### Comparative security

#### Comparative security analysis with SeND

SeND has been proposed by IETF and specified by RFC 3971 to improve IPv6 link-local network. SeND introduces a new address format called ‘CGA’ to allow the host to prove that it has a unique IP address. SeND is based on SHA-1, which is already broken. Therefore, it is no longer safe to be used. However, DAD-match relies on SHA-3, which is more secure than SHA-1. Also, the message verification process in SeND is performed by doing hash calculation two times, which leads to complexity and needs extra time. DAD-match verification process, on the other hand, performs only one time, which is faster and less complex.

#### Comparative security analysis with Trust-ND

Trust-ND mechanism aims to secure the DAD process based on Trust status. Trust value calculation is performed through a combination of the result of verification and senders trust status, which is stored earlier in its Trust Neighbor Cache. Nevertheless, in case any node has recently joined the link, it will not have any trust value that it can be an attacker and send a fake NA-trust message. The trust-ND mechanism is based on the SHA-1 hash function, which has already been broken and, therefore, it is no more recommended to be used.

#### Comparative security analysis with HSEC-Target-DAD

HSEC-target-DAD utilizes the hybrid method (Hash and asymmetric encryption) to secure the tentative IP address. The proposed mechanisms change the NS and NA message types to 138 and 139, respectively, which are already taken by other ICMPv6 messages. The author suggested using FF02::8 instead of FF02::1 to exclude the attack from joining DAD process. However, FF02::8 is also taken by IS-IS for IPv6 routers. The proposed mechanism uses blacklist when receiving a fake NA message. Based on this procedure, the attacker may use another legitimate node MAC address and send a fake NA-h message. Therefore, HSEC-target-DAD will induce DoS attack by preventing legitimate nodes from joining DAD process. The HSEC-target-DAD is vulnerable to a high collision attack probability because of using 64-bits only of the hash values (512-bits).

## Experiments and evaluation of proposed DAD-match security technique

The proposed technique DAD-match has been implemented based on Java programing language. The experiments were carried out on the same machine with Intel (R) Core (TM) 2 Quad CPU Q8400 @ 2.67GHz and using Windows 10 Pro (64-bit) Operating System. The experiments were performed to measure the performance of the proposed technique DAD-match and compare the results with the Standard-DAD process, SeND, Trust-ND, and HSEC-Target-DAD mechanisms. This is to ensure that DAD-match functions in an appropriate manner and the proposed technique meets security requirements. The experiments’ scenarios are carried out using two common tools named (dos-new-ipv6 attacker tool and Scapy attacker tool) which have been used by recent research communities such as [6], [18], [28], [36]. Further, DAD-match technique is compared with other mechanisms based on the processing time of Sender and Receiver to generate and verify NS and NA messages during the DAD process in IPv6 link-local-network.

### Attack scenarios

#### dos-new-ip6 attacker tool

The aim of this scenario is to measure the performance and the ability of DAD-match to secure DAD process in IPv6 link-local network during attacker existing. The experiment used dos-new-ipv6 attacker tool which available in THC IPv6 [37]. This attack aims at keeping sending a fake NA message to each DAD’s NS message. Since the target node does not have any verification mechanisms, the DAD process will fail and, accordingly, prevent the target node from joining the IPv6 network.

The experiments were repeated 20 times on Standard-DAD and DAD-match. To measure each mechanism’s ability to prevent attacks during the DAD process, Eq (6) is used:

$$DADSR=1-\frac{F}{N}$$ (6)

Where *DADSR* is DAD success rate, *N* is the number of DAD times, and *F* is DAD times failed. Based on DADSR definition, it is concluded that if DADSR is 1, this means that the attack is completely prevented. However, if DADSR is 0, this indicates that the mechanism cannot prevent attacks. DADSR can, therefore, be used to measure the ability of each mechanism.

**Fig 8:**
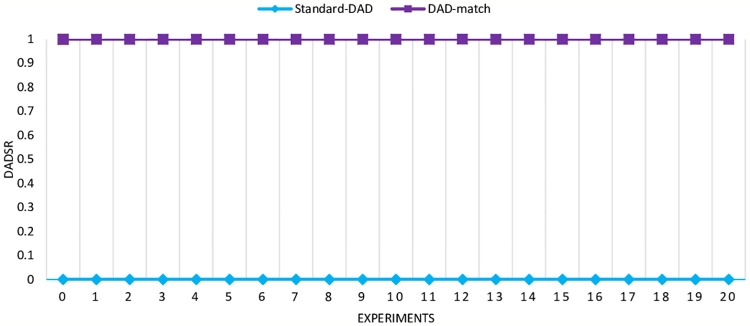
DoS attack during DAD process on Standard-DAD and DAD-match security technique.

Fig 8 illustrates the experimental results. The obtained results revealed that Standard-DAD does not have the ability to prevent attacks. When DADSR is 0, this made the DAD process fail, but when DADSR is 1 for DAD-match, this indicates that attacks are totally prevented by the proposed DAD-match security technique.

#### Scapy attacker tool

Scapy is a packet manipulation tool for modifying messages, i.e., NS and NA messages [38]. Scapy is used to build the same message based on the mechanism that is used during the experiment such as NS-trust and NA-trust for Trust-ND mechanism and NS-SeND and NA-SeND for SeND mechanism. Further attackers may modify the IP and MAC address to launch a DoS attack to disturb the DAD process in IPv6 link-local network. Therefore, Scapy has been used for this mission. The experiment was repeated 20 times on each mechanism including DAD-match technique using Eq (4) to measure DAD success rate. Table 3 presented the experiment results.

**Table 3:** DADSR comparison between DAD-match and existing mechanisms during DoS-spoofing IP address attack on DAD process.

Mechanism	Number of Experimental runs	Success DAD	Failure DAD	DADSR
SeND	20	20	0	1
Trust-ND	20	0	20	0
HSEC-TARGET-DAD	20	0	20	0
DAD-match	20	20	0	1

The experimental results exhibited that SeND mechanism and the proposed technique DAD-match able to prevent attacks. Whereas, the other existing mechanisms i.e. Trust-ND and HSEC-Target-DAD have failed to secure the DAD process. Although, SeND mechanism prevent DoS attack during DAD, however, it needs high processing time for verifying NS-SeND and NA-SeND messages, as explained in below section.

### Processing time analysis

An analysis of the processing time for generating and verifying NS and NA messages at sender and receiver hosts is given in this section. Moreover, a comparison of the obtained results with all the existing mechanisms’ results is made to prove the efficiency of DAD-match security technique.

#### Generating NS and NA messages at the sender host

Both NS and NA messages will be sent by the sender host to complete the DAD process in IPv6 link-local network. For DAD-match technique, each sender host should generate a DAD*match* option with its required fields and attach it to each of the NS and NA messages. The measurement of processing time *PT* in the sender host is done by subtracting ending time *Et* with starting time *St* of the message generation process as shown below in Eq (7):

PT=Et-St (7)

**Fig 9:**
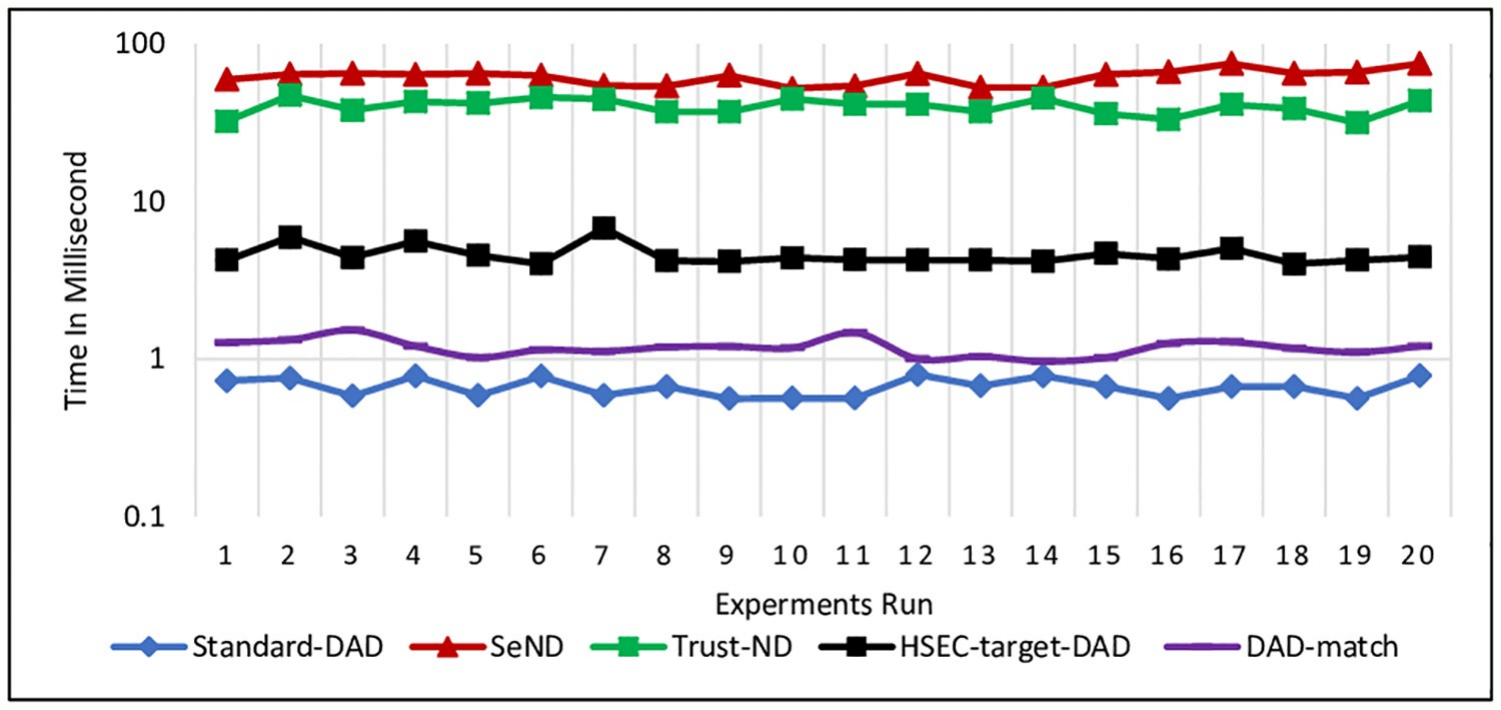
Processing time for generating NS message at the Sender node.

**Fig 10:**
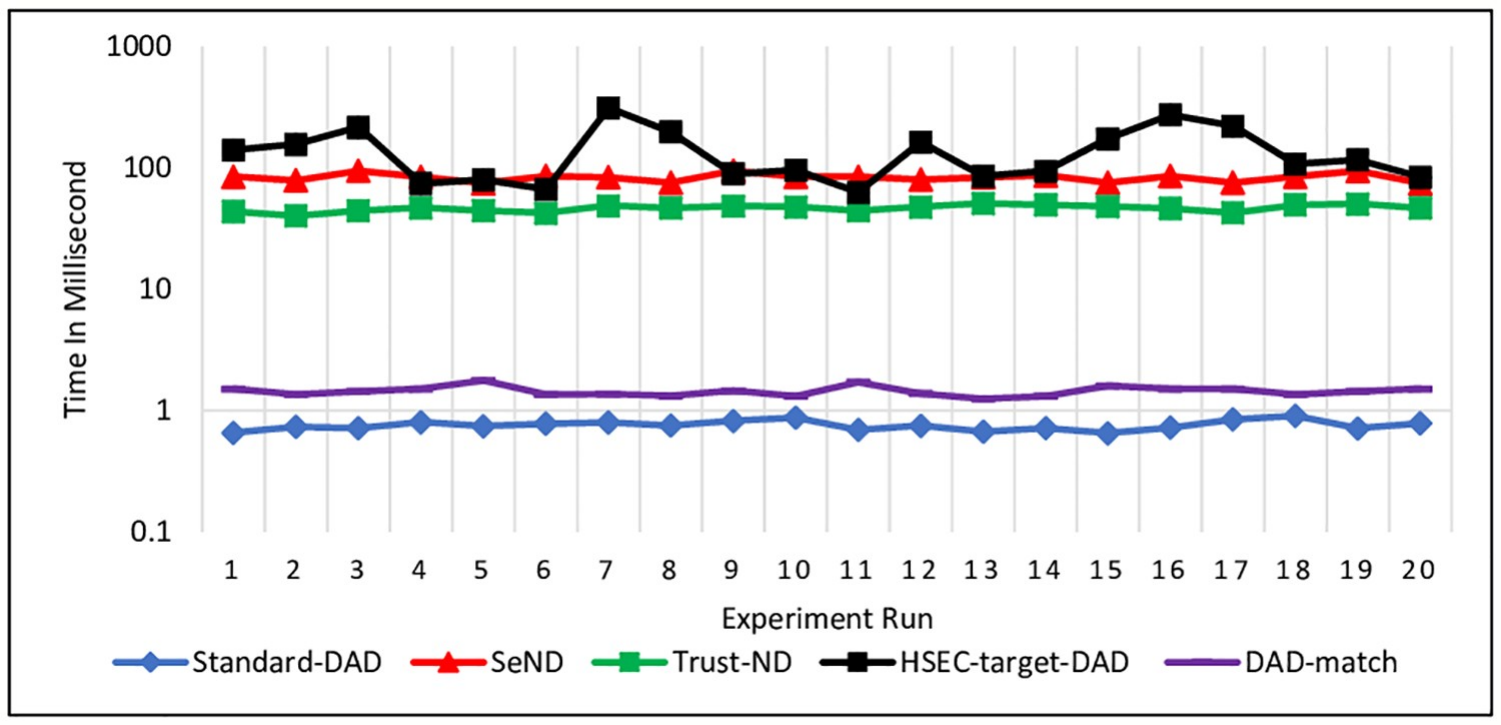
Processing time for NA message at the Sender node.

The experiment was conducted 20 times for each message i.e. NS and NA messages. The processing time to generate NS and NA messages is illustrated in Figs 9 and 10, respectively.

Based on the experiment results, it is obvious that all the existing mechanisms needed much time to generate the NS and NA messages. Standard-DAD certainly consumes less time to generate NS and NA messages since it does not have any additional security options that need to be generated such as Trust option or DAD*match*.

#### Verifying NS and NA messages at the receiver host

Each of the hosts receives a message about whether NS or NA messages should perform a message verification to prevent any fake messages that can disturb DAD process based on the mechanism strategy. For DAD-match security technique, the receiver host needs to check the existence of DAD*match* option first, then proceeds with the verification by calculating the hash values. Eq (5) is used to measure the processing time and the experiment was repeated 20 times on each message.

**Fig 11:**
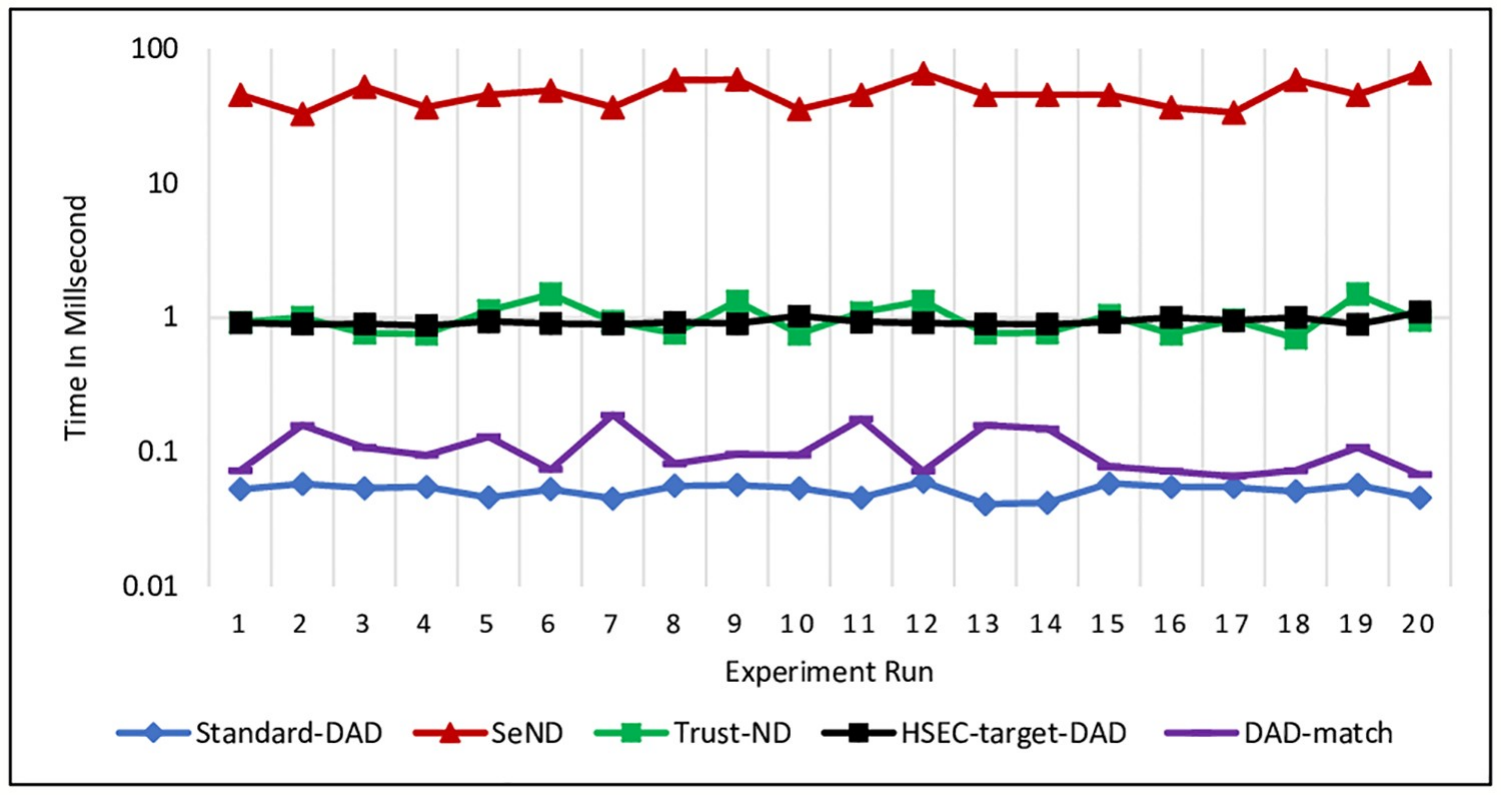
Processing time for verifying NS message at the Receiver node.

**Fig 12:**
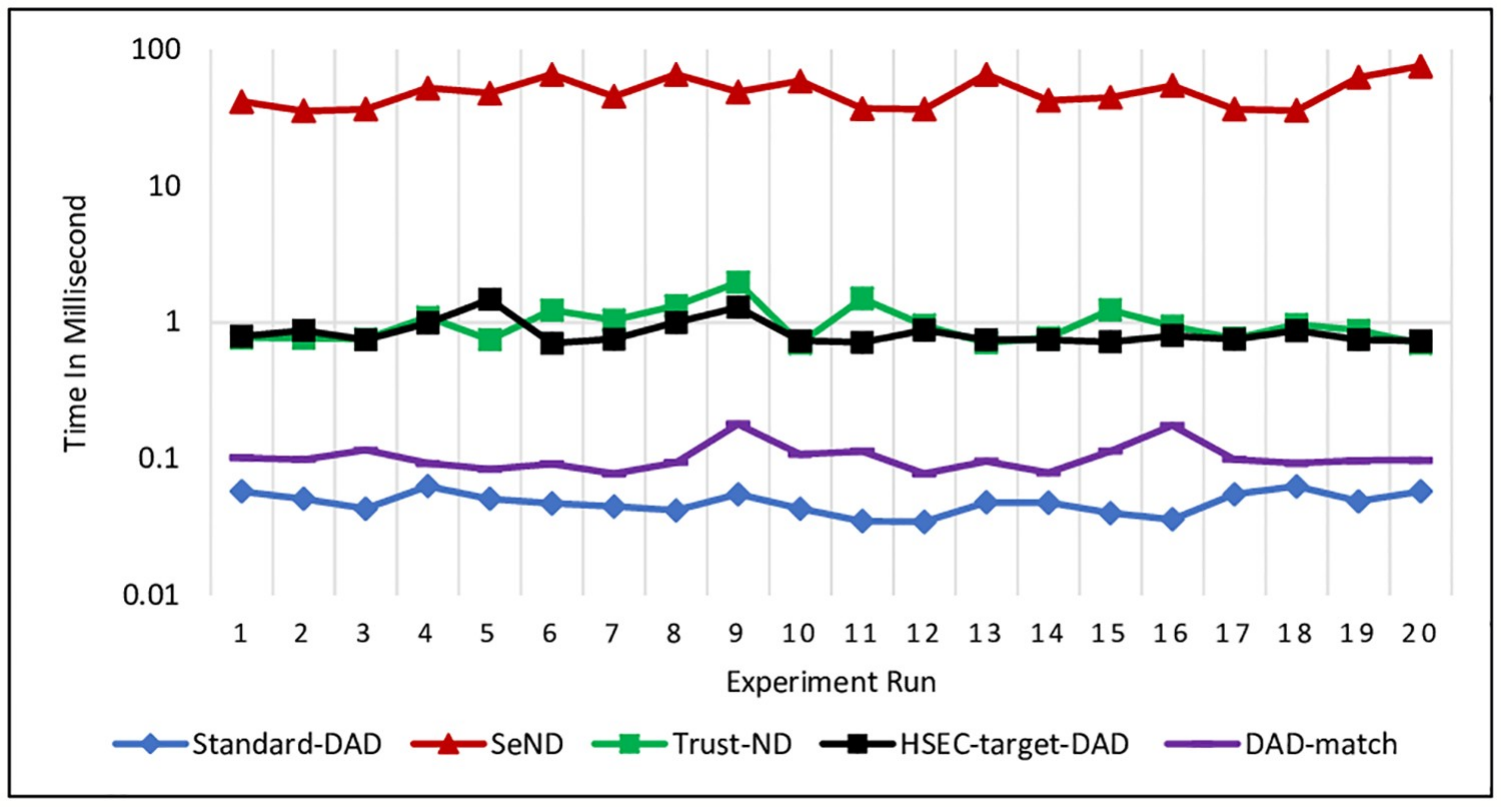
Processing time for verifying NA message at the Receiver node.

Figs 11 and 12 show the processing time for verifying NS and NA messages, respectively. The obtained results revealed that DAD-match technique consumes less processing time compared with other existing mechanisms. Further, Standard-DAD consumes less processing time compared to proposed technique DAD-match, because it does not have any security verification.

Table 4 illustrates the mean, standard deviation (STDVE), and overhead for generating and verifying NS and NA messages, respectively. The overhead estimation is carried out by placing the Standard-NS message average processing time as a baseline. Accordingly, the processing time of the mechanisms’ messages is calculated.

**Table 4:** Processing time for generating and verifying NS and NA messages.

NDP Messages	Processing Time (ms)			
	Generating Message		Verifying Message	
	NS	NA	NS	NA
Standard-DAD				
Mean	0.6692	0.7546	0.0521	0.0482
STDVE	0.0905	0.0699	0.0057	0.0086
Overhead	Baseline	Baseline	Baseline	Baseline
SeND				
Mean	62.4186	83.2133	46.9062	47.3722
STDVE	6.8534	6.3387	10.29262	15.31615
Overhead	61.7494	82.4587	46.8541	47.324
Trust-ND				
Mean	40.2823	46.4487	0.9855	0.9898
STDVE	4.63392	2.9134	0.2535	0.3256
Overhead	39.6130	46.6357	0.9334	0.9416
HSEC-Target-DAD				
Mean	4.6202	140.5742	0.9334	0.8531
STDVE	0.7200	72.2655	0.0578	0.2019
Overhead	3.9510	139.8196	0.8813	0.8049
DAD-match				
Mean	1.2050	1.4453	0.14495	0.0929
STDVE	0.1178	0.13447	0.01819	0.0137
Overhead	0.5357	0.6907	0.09285	0.0447

**Fig 13:**
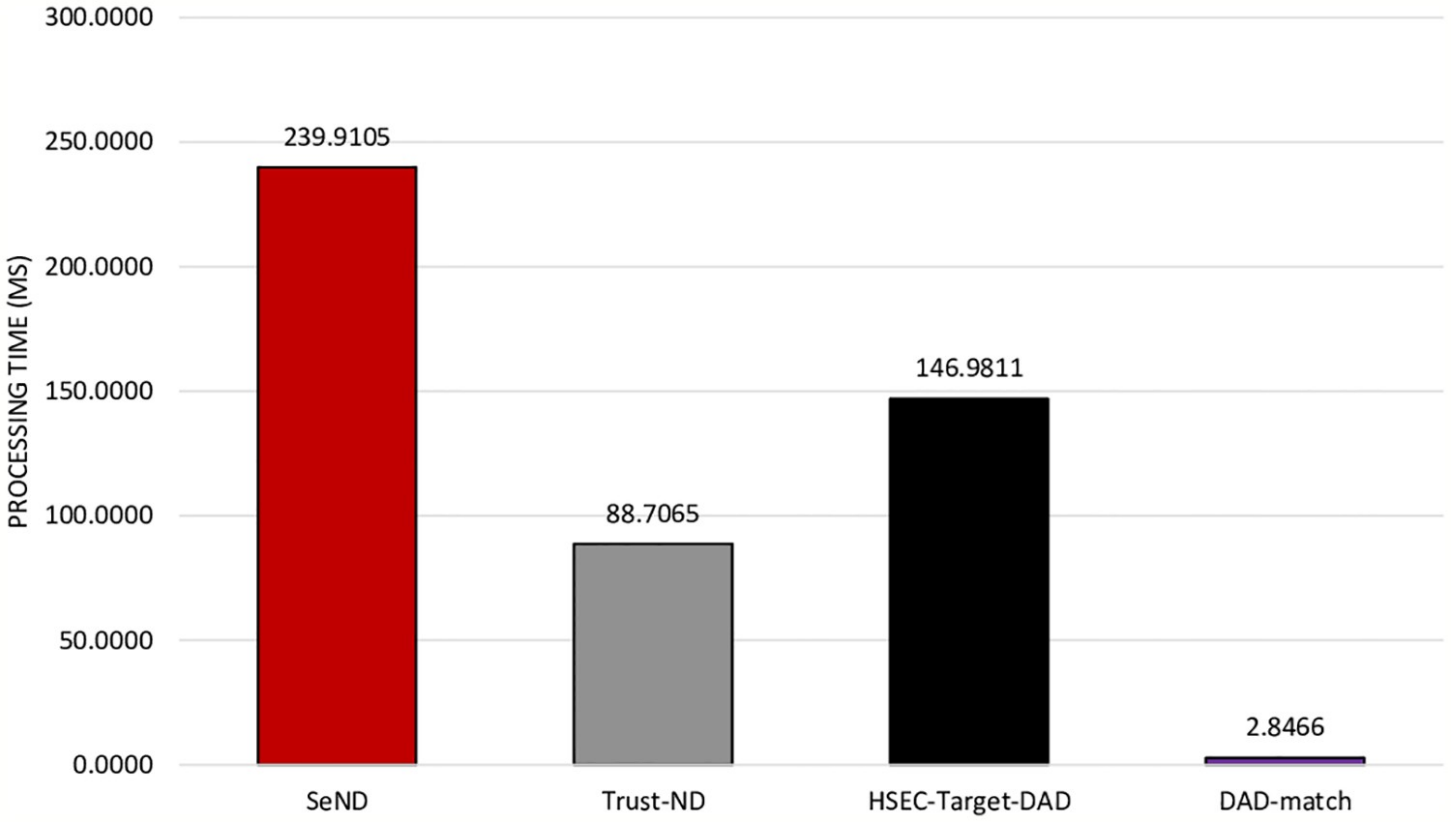
Comparative results of DAD-match security technique with other existing mechanisms in terms of total processing Time.

Based on Table 4, it is noticeable that the existing mechanisms’ processing time such as SeND, Trust-ND, and HSEC-Target-DAD is considerably high compared with the standard process of Standard-DAD. Whereas, DAD-match technique consumes less processing time compared with other existing mechanisms. Based on the overall results, the proposed technique can clearly reduce the level of complexity issues, i.e., the processing time of NS and NA messages’ generation and verification between hosts during DAD process in IPv6 link-local network. Fig 13 shows comparative results of DAD-match security technique with other existing mechanisms in terms of total processing Time.

## Discussion

When the new node implements the DAD-match security technique, it will validate all the incoming messages, that is, NS-match and NA-match messages. It checks the DAD*match* option existence in the first place. If the DAD*match* option is missing, the node should discard the message. If the DAD*match* exists, the algorithm will proceed with the verification process by matching the hash values followed by Nonce, which indicates a correct response message. If the hash values and Nonce match, the duplicate IP address occurs, and the new node needs to regenerate a new IP address and repeat the DAD-match process. In this case, the new node will be able to validate the incoming message without the need for any third-party device and successfully configure its IP address.

Based on the above experimental scenarios and the obtained results, it is obvious that DAD-match technique improved DAD process in IPv6 link-local network in terms of the processing time and effectiveness so that a DoS attack during DAD process is completely prevented. The results demonstrated that DAD-match technique consumes less processing time for both NS and NA messages compared with SeND mechanism and other existing mechanisms. In addition, DAD-match technique is completely able to secure the DAD process, and it allows all the nodes to securely configure their IP addresses.

## Conclusion and future work

Duplicate Address Detection is one of the NDP processes to detect the unique tentative IP address on IPv6 network using two of NDP messages: NS and NA messages. Each node needs to perform the DAD process before joining the IPv6 network as it is very important to secure DAD process. However, the DAD process does not have any verification mechanism to validate incoming messages. Therefore, DAD process is vulnerable to DoS attack. Any attacker on the link can keep sending a fake NA message as a response to NS message and claim the tentative IP address in NS message has been used by another node. As a result, the victim will not be able to verify the unfitness of tentative IP address, which prevents it from joining the IPv6 network.

DAD-match security technique is proposed to prevent DoS attack during DAD process in IPv6 link-local network by hiding the tentative IP address using SHA-3 hash function algorithm. Security analysis and comparative analysis were carried out on the existing mechanisms like SeND, Trust-ND, and HSEC-Target-DAD. The implementation is carried out in two scenarios to measure the performance of DAD-match technique and compare the results with the existing mechanisms’ results. The obtained results revealed that the DAD-match technique consumes less processing time. The technique is more efficient compared with SeND and other mechanisms. DAD-match technique resists various types of attacks like collision and brute-force attacks. Accordingly, it is concluded that the DAD-match technique can efficiently prevent the DoS attack during the DAD process. In the context of this study, DAD-match technique is implemented on a small area IPv6 network. For further studies, the authors recommend that the DAD-match technique is implemented and tested on a large area IPv6 network.
